# The integral role of lifestyle in the prevention and management of hypertension and associated cardiometabolic and cognitive disorders: a review

**DOI:** 10.3389/fendo.2025.1682814

**Published:** 2025-12-01

**Authors:** Qian Wang, Dan Wu, Yan Huang

**Affiliations:** 1 Health Management Center, General Practice Medical Center, West China Hospital, Sichuan University, Chengdu, China; 2 West China Hospital, Sichuan University, Chengdu, China; 3 Research Laboratory for Prediction and Evaluation of Chronic Diseases in the Elderly, National Clinical Research Center for Geriatric Diseases, Chengdu, China; 4 General Practice Research Institute, West China Hospital, Sichuan University, Chengdu, China

**Keywords:** hypertension, lifestyle intervention, cardiovascular disease, cognitive decline, prevention, management, cardiometabolic syndrome, digital health

## Abstract

Hypertension represents a paramount global health challenge, intricately linked to cardiovascular, metabolic, and cognitive morbidity. This narrative review provides a critical synthesis of current evidence, anchored by a systematic literature search, to delineate the integral role of comprehensive lifestyle interventions in the prevention and management of hypertension and its complications. Our analysis demonstrates that evidence-based, multidimensional strategies—including dietary modifications (e.g., DASH and Mediterranean diets), regular physical activity, structured weight management, and stress reduction—effectively lower blood pressure, improve metabolic parameters, and attenuate target organ damage. These non-pharmacological approaches act synergistically with antihypertensive drug therapy and can be personalized through digital health technologies. The findings underscore that embedding structured lifestyle medicine into clinical practice and public health policy is an indispensable, cost-effective strategy for alleviating the global burden of hypertension.

## 1 Introduction

Hypertension, or high blood pressure, is a significant global health issue, closely associated with cardiovascular, metabolic, and cognitive disorders. High blood pressure remains one of the most prevalent and impactful non-communicable diseases (NCDs) globally, significantly contributing to morbidity and mortality worldwide. Its insidious nature often leads to delayed diagnosis and treatment, silently predisposing individuals to a myriad of severe health complications (1, 2). The rising global prevalence of hypertension poses an immense burden on healthcare systems and public health initiatives, necessitating robust and sustainable strategies for prevention and management. Central to these strategies is the recognition of lifestyle as a powerful, modifiable determinant of health, capable of significantly influencing the trajectory of hypertension and its associated comorbidities.

The intricate interplay between hypertension and other chronic conditions, notably cardiovascular diseases (CVDs), metabolic syndrome, chronic kidney disease (CKD), and cognitive decline, underscores the imperative for a holistic approach to patient care. There is consistent evidence of a causal association between cardiovascular risk factors and the development of hypertension, establishing a vicious cycle of deteriorating health (3). Moreover, conditions such as obesity (4), diabetes, and dyslipidemia frequently co-exist with hypertension, forming the complex constellation known as cardiometabolic syndrome (5, 6). Beyond its well-established cardiovascular ramifications, hypertension is increasingly recognized as a critical risk factor for cognitive impairment and dementia, with lifestyle factors playing a substantial role in either mitigating or exacerbating this risk (7–9).

This review aims to provide a broad and insightful overview of the current scientific literature concerning the integral role of lifestyle interventions in addressing hypertension and its associated cardiometabolic and cognitive disorders. First, we will meticulously detail the burden of hypertension and its interconnectedness with various health conditions. We will then explore the diverse range of evidence-based lifestyle strategies constitutes the foundation of both primary prevention and effective therapeutic management. We will also explore the evolution of these interventions, incorporating modern, integrated approaches and technological advancements that enhance their reach and efficacy. Through this critical analysis, the review aims to consolidate the understanding of the vital role of lifestyle in a holistic health paradigm.

## 2 Methods

To ensure a comprehensive and structured synthesis of the current evidence, a systematic literature search strategy was designed and executed. The primary objective was to identify high-quality, recent publications relevant to the role of lifestyle interventions in hypertension and its associated disorders.

### 2.1 Search strategy

A focused electronic literature search was performed using the PubMed database. The search was restricted to articles published from January 1, 2020, to June 30, 2025, to ensure the inclusion of the most recent and relevant evidence. The search strategy combined key Medical Subject Headings (MeSH) terms and free-text keywords related to the core themes of the review.

The main search blocks included:

Block 1: (“Hypertension”[Mesh] OR “High Blood Pressure”)

Block 2: (“Life Style”[Mesh] OR “Diet”[Mesh] OR “Exercise”[Mesh] OR “Physical Activity” OR “Weight Management”)

Block 3: (“Cardiovascular Diseases”[Mesh] OR “Cognitive Dysfunction”[Mesh] OR “Dementia”[Mesh] OR “Renal Insufficiency, Chronic”[Mesh] OR “Metabolic Syndrome”[Mesh])

Block 4: (“Prevention and Control”[Subheading] OR “Therapeutics”[Mesh] OR “Management”)

Block 5 (Study Design Filter): (“randomized controlled trial”[pt] OR “controlled clinical trial”[pt] OR “systematic review”[pt] OR “meta-analysis”[pt] OR “prospective studies”[MeSH] OR (prospective[tiab] AND cohort[tiab]))

Blocks 1–4 were combined using ‘AND’, and the resulting set was then combined with Block 5 using ‘AND’ to focus on specific study designs.

### 2.2 Selection and eligibility criteria

The identified records were screened for relevance based on titles and abstracts, followed by a full-text assessment of potentially eligible articles.

Inclusion Criteria: We included randomized controlled trials (RCTs), prospective cohort studies, systematic reviews, and meta-analyses that investigated at least one lifestyle intervention (diet, exercise, weight loss, etc.) for the prevention or management of hypertension and its cardiometabolic or cognitive complications.

Exclusion Criteria: We excluded studies published in languages other than English, preclinical studies, editorials, letters, commentaries, and studies without original data.

### 2.3 Data synthesis

Given the broad, multi-domain scope of this review, a formal meta-analysis was not feasible. Instead, a narrative synthesis approach was adopted. The selected evidence was organized into thematic sections to provide a coherent and critical overview of the current scientific landscape.

## 3 The burden of hypertension and its interconnectedness with cardiometabolic and cognitive health

Hypertension poses a heterogeneous and substantial burden on global public health, with its prevalence and control rates demonstrating significant disparities across geographic, socioeconomic, and demographic lines. While trends have improved in some high-income countries, a pronounced rise is observed across East and South Asia, Oceania, and sub-Saharan Africa. These regions, along with many low- and middle-income countries, are further challenged by low rates of treatment and effective control. Beyond geography, the burden of hypertension is not uniformly distributed by gender or residence. Men generally exhibit higher blood pressure levels than women in younger populations, a gap that narrows with age, though this pattern exhibits regional variation. Furthermore, complex disparities exist between rural and urban areas, often mediated by factors such as socioeconomic status and educational attainment. Access to healthcare resources is crucial (10). Hypertension is often referred to as a “silent killer” due to its asymptomatic nature in the early stages, leading to delayed diagnosis and an increased risk of end-organ damage (1). It has been identified as the most significant modifiable risk factor for cardiovascular disease (CVD) events, including coronary heart disease, heart failure, and stroke, as well as mortality. The relationship between cardiovascular risk factors and hypertension is not just correlational; it is fundamentally causal, with specific risk factors directly contributing to the development and progression of elevated blood pressure (3). This establishes hypertension as a central component within a broader network of cardiometabolic dysregulations. For example, the COVID-19 pandemic has been associated with an increased prevalence of metabolic syndrome, highlighting how systemic inflammation and lifestyle disruptions can exacerbate cardiometabolic risks (5). Individuals with hypertension often present with multiple cardiometabolic comorbidities, a phenomenon known as cardiometabolic multimorbidity. Encouragingly, adopting healthy lifestyle habits has been shown to significantly lower the risk of developing this multimorbidity in hypertensive patients, emphasizing the protective role of lifestyle (6). Ideal cardiovascular health, which includes optimal blood pressure, lipid profiles, glucose metabolism, and lifestyle factors, is profoundly associated with a reduced risk of various diseases, further emphasizing the interconnectedness of these health domains (11).

Beyond its direct impact on the cardiovascular system, hypertension is a critical determinant of cognitive health and a significant risk factor for cognitive impairment, including vascular dementia and Alzheimer’s disease. Hypertension has been identified as a significant risk factor for cognitive decline, particularly in middle age, with notable correlations observed between. Hypertension and cognitive impairment, as well as brain morphological changes in later life, are of concern. Blood pressure variability (BPV) has been identified as an independent risk factor for cognitive decline. Hypertension is a condition that has the potential to cause a series of detrimental effects on bodily functions. These effects include vascular damage, inflammation, neurovascular dysfunction, and disruption of the blood-brain barrier (BBB). Collectively, these factors can significantly impact brain health, resulting in neuronal damage and subsequent cognitive decline. Furthermore, hypertension has been demonstrated to exacerbate cognitive decline through mechanisms such as affecting cerebral blood flow, cerebrospinal fluid mobility, and cerebral small vessel disease (CSVD). It is important to note that, in contrast to the prevailing traditional view, which considers Alzheimer’s disease (AD) to be purely a neurodegenerative disorder, contemporary perspectives emphasize the significant role of vascular brain lesions in AD, with the majority of patients exhibiting a combination of pathological features. Additionally, hypertension has been shown to exacerbate the progression of Alzheimer’s disease (AD) pathology through the promotion of the accumulation of β-amyloid (Aβ) and phosphorylated tau protein (p-tau) (12). A substantial proportion of dementia cases, potentially up to one-third, are considered preventable through the modification of lifestyle and vascular risk factors (8). Indeed, modifiable risk factors account for approximately 50% of dementia cases in populations like Canada, underscoring the immense potential for prevention through targeted interventions (7). Some studies have shown that blood pressure treatment may help reduce the risk of cognitive decline, particularly in individuals who start treatment in middle age. Intensive blood pressure lowering, for instance, has been demonstrated to reduce the risk of cognitive impairment, indicating a direct link between effective hypertension management and cognitive health. Tension management and brain health (9). Effective blood pressure control can slow the progression of cerebral small vessel disease (13). Furthermore, lifestyle interventions have been shown to improve cerebrovascular function, thereby potentially mitigating cognitive decline (14). Tools like the LIBRA score, which incorporates lifestyle factors and medical history, can predict cognitive function, highlighting the cumulative impact of various health determinants on brain health (15). The bidirectional association between hypertension and cognitive function underscores the importance of early and sustained intervention.

The pervasive impact of hypertension extends to a spectrum of other severe health outcomes. Stroke: Hypertension is the leading risk factor for stroke. A common dietary pattern of low vegetable intake significantly increases the risk of stroke in hypertensive patients (16). Both primary and secondary stroke prevention strategies heavily rely on blood pressure control and lifestyle modifications (17, 18). Digital platforms are increasingly utilized to support stroke prevention efforts, enhancing patient engagement and adherence (19). Atrial Fibrillation (AFib): Lifestyle interventions have shown promise in reducing the risk of atrial fibrillation, a common cardiac arrhythmia that significantly increases stroke risk (20). Even with optimal medical therapy, approximately 30% of residual stroke risk in AFib patients is attributable to modifiable factors, emphasizing the continued relevance of lifestyle interventions (21). Heart Failure (HF): Elevated blood pressure is a primary driver of heart failure development. Maintaining a healthy lifestyle has been shown to significantly reduce the risk of heart failure (22). Furthermore, conditions such as high serum sodium levels have been linked to an increased risk of heart failure (23). In South Asian populations, the rising burden of heart failure is strongly associated with diabetes, which is another cardiometabolic condition closely linked to hypertension (24).For individuals who have received a heart transplant, tailored prevention and rehabilitation programs that incorporate lifestyle elements are crucial for long-term health (25). Chronic Kidney Disease (CKD): Hypertension and CKD share a strong bidirectional relationship, where each condition can cause or exacerbate the other (26). Dietary interventions, such as plant-based diets, can help to manage kidney disease (27), and the Dietary Approaches to Stop Hypertension (DASH) diet has been shown to reduce the risk of CKD (28). The co-exposure to organic pollutants and diabetes further exacerbates the risk of kidney disease (29). Cancer: Although the primary focus of hypertension research is cardiovascular health, there are also links to cancer. Blood pressure-lowering treatment generally does not significantly increase the risk of cancer, which is reassuring for those undergoing long-term antihypertensive therapy (30). However, Western lifestyle patterns have been linked to an increased burden of gastrointestinal cancers (31), and specific lifestyle factors such as smoking and obesity increase the risk of renal cancer (32). Air pollution has also been positively correlated with an increased risk of colorectal cancer (33), highlighting the importance of broader environmental determinants of health. Lifestyle interventions can also modulate cancer metabolism (34). Pregnancy-Related Conditions: Hypertension during pregnancy, including gestational hypertension and pre-eclampsia, significantly increases the long-term cardiovascular risk for the mother (34). Comprehensive interventions are critical for managing hypertension and diabetes during pregnancy (35). Lifestyle interventions are also effective in reducing the risk of gestational diabetes (36). International guidelines for the management of hypertension management show both similarities and differences, emphasizing the need for adaptable approaches (37). Pre-pregnancy health optimization is vital for reducing adverse pregnancy outcomes (38), and long-term management strategies are essential for women with pregnancy-induced hypertension (39). Other Conditions: The interconnectedness extends to conditions like Polycystic Ovary Syndrome (PCOS), which increases the risk of cardiovascular disease (40). Post-Traumatic Stress Disorder (PTSD) has been shown to increase the incidence risk of hypertension (41), highlighting the role of mental health in cardiovascular well-being. Gout, traditionally managed through diet, also necessitates an approach that considers metabolic health, as obesity is a major preventable gout risk factor (42, 43). Childhood obesity and specific childhood cancers can also predispose individuals to health challenges in adulthood, underscoring the importance of early lifestyle interventions (44, 45).

**Figure 1:**
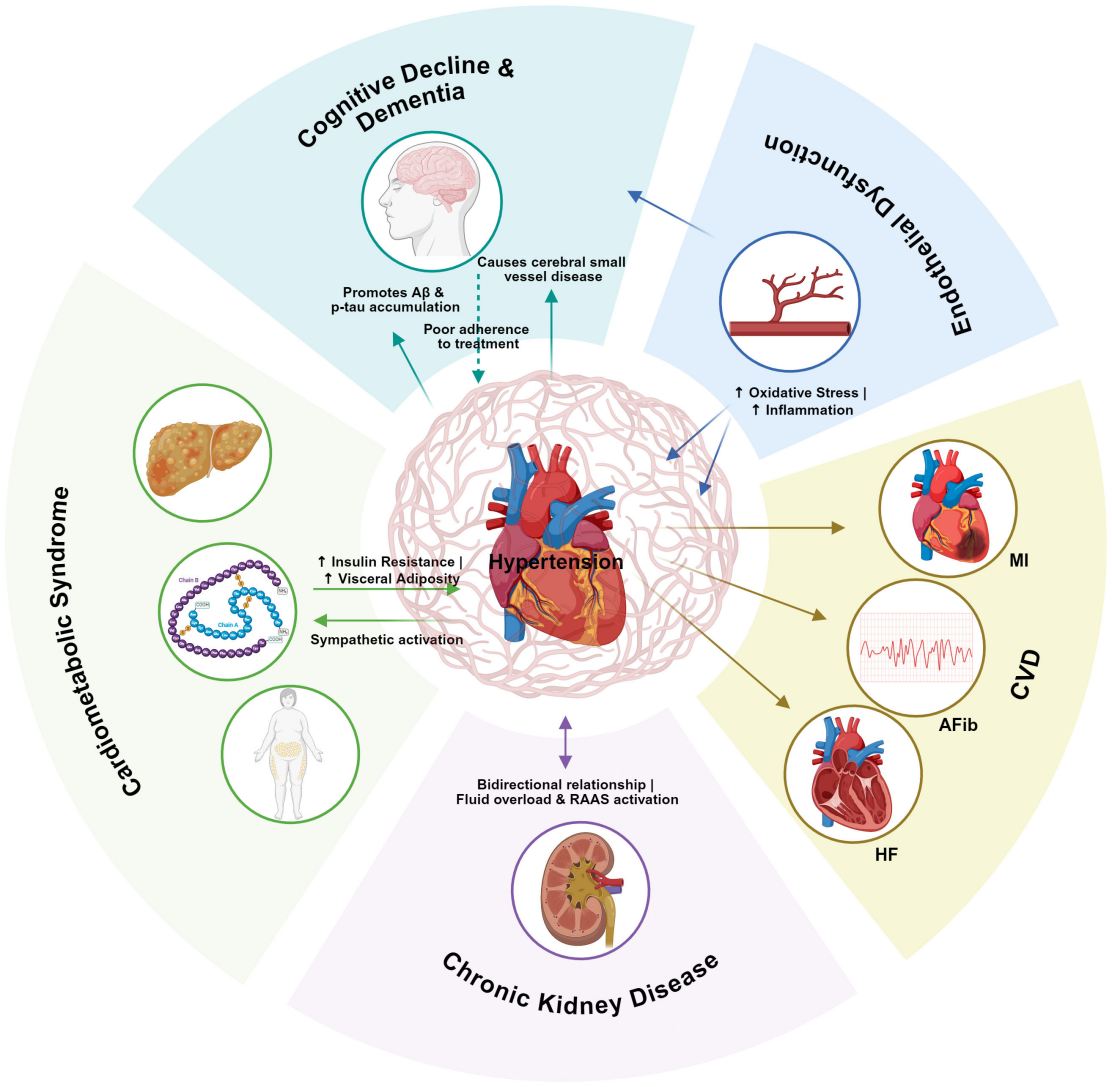
The interconnected web of hypertension and its cardiometabolic and cognitive comorbidities. This schematic illustrates thecomplex bidirectional relationships between hypertension (center) and its major endorgan complications. Solid arrows indicate direct pathological effects, while the dashed arrow represents a behavioral consequence. Key pathophysiological pathways, such as endothelial dysfunction, oxidative stress,and inflammation,underpin these connections (see Section 3 for details). Aß, ß-amyloid; p-tau, phosphorylated tau; CVD, cardiovascular disease; HF, heart failure; AFib, atrial fibrillation;MI, myocardial infarction; RAAS, renin-angiotensin-aldosterone system.

The intricate and bidirectional relationships between hypertension and its associated complications are complex and multifaceted. To visually summarize these interconnections and provide a conceptual framework for understanding the systemic nature of hypertension, **Figure 1** illustrates the interconnected web linking hypertension with cardiometabolic and cognitive comorbidities.

As depicted in **Figure 1**, hypertension rarely occurs in isolation but rather functions as a central node within a complex network of interrelated conditions. This interconnectedness underscores the imperative for holistic management strategies that address not only blood pressure control but also the broader spectrum of cardiometabolic and cognitive health.

## 4 Lif*est*yle interventions: foundations of prevention and management

Lifestyle interventions form the cornerstone of both primary prevention and therapeutic management for hypertension, often serving as the first line of defense and a vital adjunct to pharmacotherapy. Their efficacy stems from addressing fundamental physiological processes influenced by daily habits. Non-pharmacological interventions are widely recommended in hypertension guidelines, emphasizing their foundational role (46, 47). Lifestyle interventions have been demonstrated to be effective in addressing a range of medical concerns, with their efficacy being sustained over time. In contrast, the benefits of pharmaceutical treatment have been shown to be limited in duration and scope. The cumulative benefits of a healthy lifestyle alongside antihypertensive medication have been shown to significantly reduce mortality rates (48).

**Figure 2:**
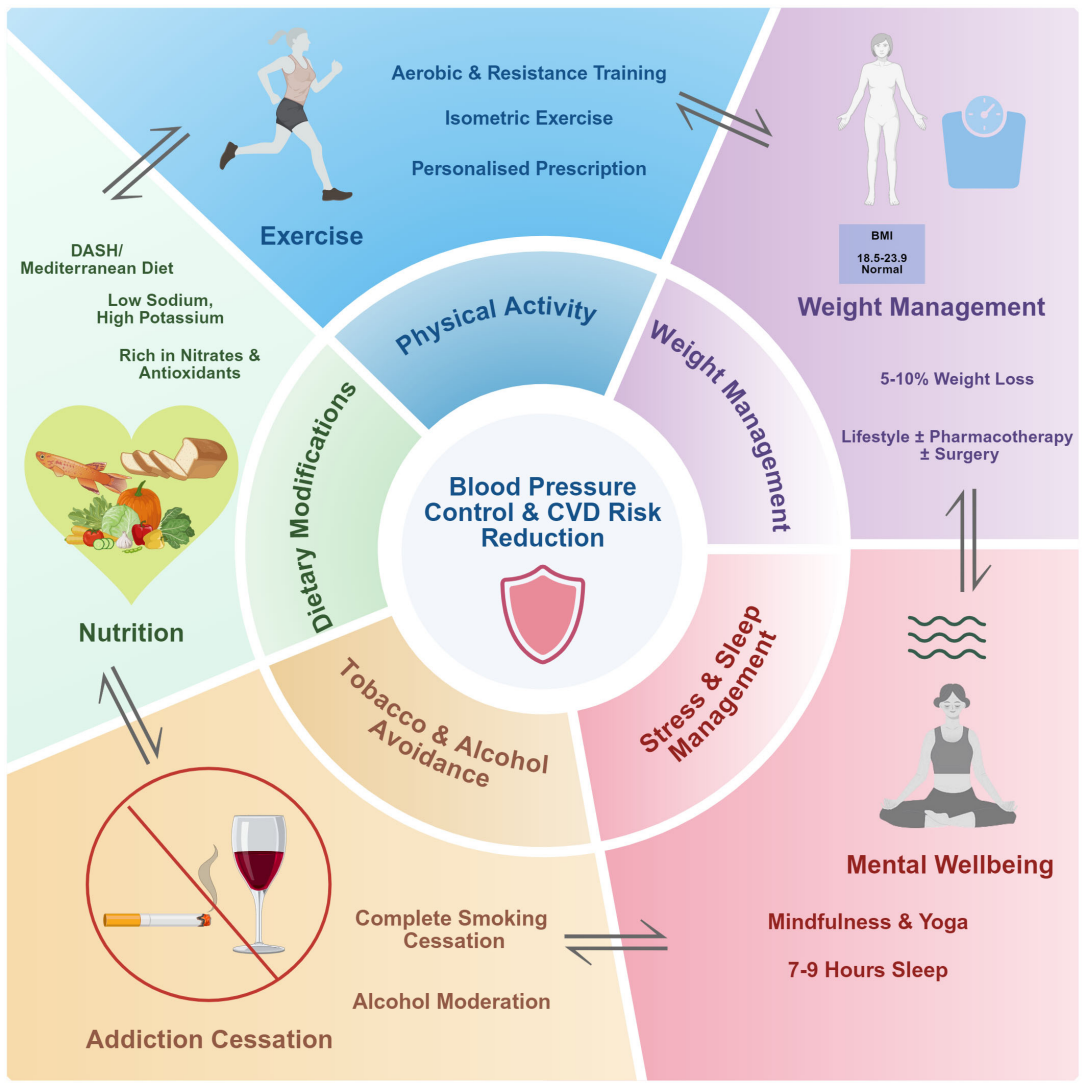
A multimodal and synergistic framework for lifestyle intervention in hypertension. The achievement of optimal blood pressure control and cardiovascular risk reduction (center) is best accomplished through the concurrent and synergistic application of five core lifestyle domains. Each domain contributes through distinct yet overlapping physiological mechanisms. The bidirectional arrows between domains emphasize that interventions in one area (e.g., exercise) can positively influence adherence and outcomes in others (e.g., weight management and stressreduction), creating a whole that is greater than the sum of its parts (see Sections 4 & 6).

Given the multifactorial pathophysiology of hypertension, effective management requires a comprehensive approach that targets multiple physiological pathways simultaneously. **Figure 2** presents a multimodal framework for lifestyle intervention, highlighting the five core domains and their synergistic interactions in achieving blood pressure control and cardiovascular risk reduction.

The integrative framework shown in **Figure 2** emphasizes that optimal hypertension management extends beyond isolated recommendations to encompass coordinated interventions across multiple lifestyle domains. The following sections will critically examine the evidence base supporting each of these core components, beginning with dietary strategies.

### 4.1 Dietary strategies

Dietary patterns play a pivotal role in modulating blood pressure and overall cardiometabolic health. A well-structured diet can prevent the onset of hypertension, aid in its management, and mitigate associated complications. DASH Diet: The Dietary Approaches to Stop Hypertension (DASH) diet is perhaps the most widely recognized and evidence-based dietary intervention for hypertension. Rich in fruits, vegetables, whole grains, and low-fat dairy, while being low in saturated fat, cholesterol, and total fat, the DASH diet is highly effective in lowering blood pressure (49). A meta-analysis evaluating the impact of DASH diet adherence on hypertension risk found that high adherence to the DASH diet was associated with lower hypertension risk compared to low adherence. This suggests that lifestyle changes should be initiated early, even in individuals with normal blood pressure (50). Beyond blood pressure control, the DASH diet has been specifically linked to a reduced risk of chronic kidney disease (CKD) (28). While generally highly effective, its blood pressure-lowering effect may be limited in patients with diabetes, suggesting the need for individualized approaches in comorbid conditions (51). Plant-Based and Mediterranean Diets: Plant-based dietary patterns are increasingly recognized for their health benefits. They are particularly beneficial for kidney disease patients, offering advantages in managing metabolic parameters and reducing disease progression (27). Similarly, legumes, a key component of plant-based diets, contain bioactive compounds that improve cardiovascular health (52). The Mediterranean diet, characterized by high intake of fruits, vegetables, whole grains, nuts, legumes, and olive oil, along with moderate consumption of fish and poultry, and low intake of red meat, is another well-established pattern. When combined with energy restriction, the Mediterranean diet can significantly reduce cardiovascular disease risk, including hypertension (53). Sodium and Potassium Balance: The balance between dietary sodium and potassium is a critical determinant of blood pressure. High sodium and low potassium intake are major contributors to hypertension (54). A systematic analysis found that higher baseline blood pressure was associated with greater blood pressure reduction from sodium restriction. However, it remains unclear whether baseline potassium intake levels alter the magnitude of blood pressure reduction from sodium restriction (55). When sodium intake was high, potassium supplementation exerted a greater blood pressure-lowering effect. One study found a negative correlation between higher potassium intake and higher blood pressure thresholds, and that the sodium-to-potassium ratio was a stronger predictor of blood pressure levels than sodium or potassium alone (56).Genetic factors also play a role in an individual’s blood pressure sensitivity to sodium and potassium intake, suggesting that personalized dietary recommendations might be beneficial (57). Reducing sodium intake is a fundamental recommendation in hypertension management (47). Specific Food Components and Supplements: Dietary Nitrates: Found in vegetables like leafy greens and beetroot, dietary nitrates can be converted to nitric oxide in the body, leading to vasodilation and blood pressure reduction. Research supports their role in lowering hypertension risk (58). Bioactive Peptides: These are specific protein fragments that can exert various physiological effects, including regulation of metabolic diseases, which encompass hypertension. Their role in influencing blood pressure and metabolic health is an active area of research (59). Antioxidant Vitamins: Vitamins such as A, C, and E, possessing antioxidant properties, have been hypothesized to lower blood pressure. While promising, the evidence base for direct blood pressure reduction through antioxidant vitamin supplementation alone is still evolving (60). Functional Foods: Beyond specific nutrients, certain functional foods and food waste extracts have shown promise. For instance, sweet cherry and beet waste extracts have been found to protect endothelial function, which is crucial for blood pressure regulation (61). Meta-analyses support the role of various functional foods in assisting blood pressure reduction (62). Impact on Other Conditions: Dietary considerations are also crucial for other conditions. A Western lifestyle, often high in processed foods and red meat, is associated with an increased burden of gastrointestinal cancers (32). For gout patients, dietary recommendations must holistically consider metabolic health, emphasizing foods that reduce uric acid while supporting cardiovascular well-being (42). Dietary patterns also significantly influence the progression of metabolic-associated steatotic liver disease (MASLD, formerly NAFLD), a condition often co-occurring with hypertension and insulin resistance (63). Furthermore, healthy eating habits contribute to improved glucose metabolism, which is essential for preventing and managing type 2 diabetes and its cardiovascular complications (64).

### 4.2 Physical activity and exercise

Regular physical activity is an indispensable component of a healthy lifestyle, providing profound benefits for cardiovascular health and blood pressure regulation. Blood Pressure Control: Exercise is a primary non-pharmacological intervention recommended for preventing and treating hypertension (49). Consistent engagement in physical activity improves cardiorespiratory fitness, which in turn reduces the risk of hypertension incidence (65). Personalized Exercise Prescriptions: Individualized exercise prescriptions, tailored to an individual’s health status, preferences, and goals, are more effective in lowering blood pressure. This personalized approach enhances adherence and optimizes outcomes (66). Broader Cardiovascular Benefits: Beyond direct blood pressure effects, exercise training improves cardiac conduction, reducing the risk of certain heart rhythm disorders (67) Regular physical activity also contributes to kidney protection, with exercise frequency correlated with improved renal outcomes (68). Furthermore, physical activity, alongside diet, can modulate cancer metabolism, influencing tumor growth and response to therapy (69) A healthy lifestyle, including regular exercise, significantly reduces the risk of sudden cardiac arrest (70).

Physical activity is a key lifestyle modification for the management of hypertension. Aerobic Exercise: Aerobic exercise is a form of moderate to high-intensity exercise designed to increase heart rate and metabolic rate, including brisk walking, running, swimming, cycling, and high-intensity interval training (HIIT). A meta-analysis revealed that 30 minutes of weekly aerobic exercise reduces systolic blood pressure by 1.78 mmHg and diastolic blood pressure by 1.23 mmHg. The analysis showed that the exercise has a dose-dependent effect, with the most significant reduction occurring at 150 minutes per week (71). Resistance Training: Resistance training is a form of exercise that enhances muscle strength, endurance, and muscle size by using external resistance. It not only helps increase muscle mass and bone density but also improves cardiovascular health and overall body composition (72). A meta-analysis indicates that resistance training reduces both systolic and diastolic blood pressure in elderly individuals aged 60 and over, by 6.88 mmHg and 3.37 mmHg respectively. Studies using traditional resistance equipment (free weights and machines) showed a decrease of 7.04 mmHg in systolic blood pressure and a reduction of 2.60 mmHg in diastolic blood pressure. By contrast, resistance band interventions resulted in a decrease of 2.79 mmHg in systolic blood pressure and a drop of 1.68 mmHg in diastolic blood pressure. Moderate-intensity resistance training was associated with a decrease of 6.98 mmHg in systolic blood pressure and 3.64 mmHg in diastolic blood pressure (73). Isometric resistance training (IRT): This involves sustained muscle contractions against fixed resistance, with minimal or negligible changes in muscle length. The most common form of IRT is unilateral grip squeeze exercises. Research indicates that IRT has a limited impact on 24-hour or daytime blood pressure, but significantly reduces night-time blood pressure — a finding that could help to reduce the risk of target organ damage in patients with hypertension. However, due to its lack of additional health benefits, such as controlling blood sugar and cholesterol, and the paradoxical pattern of reduced night-time blood pressure without a corresponding reduction during the day, IRT remains underutilized as an anti-hypertensive therapy (74). Isometric Exercise Training (IET): IET, which involves sustained muscle contraction without movement, has emerged as a particularly potent non-pharmacological strategy for blood pressure reduction, demonstrating significant hypotensive effects. Data from multiple systematic reviews and randomized controlled trials (RCTs) suggest that IET is more effective at reducing blood pressure than exercise regimens recommended in traditional guidelines, and is potentially as effective as standard antihypertensive drug therapy. For example, IET can reduce mean systolic blood pressure by 5–9 mmHg and diastolic blood pressure by 1–4 mmHg. IET improves blood pressure through various mechanisms, including enhanced vascular endothelial function, autonomic neurovascular regulation, and vascular remodeling. It also improves cardiac function, increasing cardiac output and reducing heart rate (HR), and enhances endothelium-dependent vasodilation in local and systemic vessels. This reduces oxidative stress and inflammatory responses (75). While IET is generally safe for most people, its effectiveness and reactions vary individually. Caution should be exercised when administering it to specific groups (e.g. patients with hypertension or cardiovascular disease), as individual health conditions and potential risks must be carefully considered. While existing studies support the blood pressure-lowering effects of IET, the small sample sizes in these studies mean that long-term efficacy and population-specific variations require further investigation. Furthermore, the long-term safety of IET, optimal training protocols and personalized prescriptions require further research.

For individuals who are beginners or those with complex health conditions, exercise should be supervised by a healthcare professional. Digital health tools, such as wearable devices and mobile applications, can support exercise monitoring and behavior change. Governments and communities should promote safe exercise environments and infrastructure to encourage public participation in physical activity. Education and policy support are essential for promoting healthy lifestyles (76).

### 4.3 Weight management

Obesity is a major modifiable risk factor for hypertension and cardiometabolic diseases, with visceral adiposity conferring greater risk than subcutaneous fat (4, 77). The pathophysiological mechanisms involve neurohormonal activation, increased renal sodium reabsorption, chronic inflammation, and physical compression of the kidneys by adipose tissue (4, 76). In children and adolescents, obesity significantly increases hypertension risk (2-fold increase in obese children, over 4-fold increase in severely obese children) (78, 79).

Therefore, effective weight management is crucial for both hypertension prevention and treatment. Weight reduction has consistently been emphasized as a key strategy for lowering blood pressure in overweight or obese individuals (80). Even moderate weight loss yields clinically significant blood pressure reductions, often diminishing or eliminating the need for antihypertensive medications. Studies indicate that maintaining a body mass index within the recommended Asian range (18.5–22.9 kg/m²) substantially lowers hypertension risk (81). Each kilogram of weight loss is associated with an average reduction of approximately 1.05 mmHg in systolic blood pressure, with particularly pronounced effects in insulin-resistant individuals (82). Controlling waist circumference (men <90 cm, women <85 cm) is a stronger predictor of blood pressure improvement than BMI alone, as visceral fat reduction directly enhances inflammation control and endothelial function (76).

#### 4.3.1 Clinical efficacy and stepwise management strategy

Given the strong association between obesity and hypertension, a structured, stepwise weight management strategy is central to achieving effective blood pressure control.

##### 4.3.1.1 Step 1: Comprehensive lifestyle intervention—first-line foundational strategy

This strategy integrates dietary modification, physical activity, and behavioral therapy. It is applicable to all overweight or obese hypertensive patients and serves as the cornerstone of treatment (83).

###### 4.3.1.1.1 Core dietary adjustments

Adhere to evidence-supported dietary patterns: Recommendation of heart-healthy diets such as DASH or Mediterranean (84).

Limit sodium intake: Restricting daily sodium to <2.3 grams (equivalent to approximately 5.8 grams of salt) reduces systolic blood pressure by 5–6 mmHg (76).

Adopt the DASH diet: This pattern emphasizes whole grains, fruits, vegetables, and low-fat dairy while reducing saturated fats, lowering systolic blood pressure by 8–14 mmHg (84). Multiple 2023 studies confirmed that adding plant-based proteins (e.g., legumes, nuts) and reducing processed meats to the traditional DASH diet further lowers systolic blood pressure by 2.4 mmHg (85).

Controlling Energy Intake: Reducing daily energy intake by 500–750 kcal with a goal of 5%-10% weight loss significantly improves blood pressure (average systolic reduction of approximately 5 mmHg) (76).

###### 4.3.1.1.2 Exercise prescription

Aerobic Exercise: ≥150 minutes of moderate-intensity aerobic activity (e.g., brisk walking, swimming) weekly can lower systolic blood pressure by 4–5 mmHg (76).

Resistance Training: Strength training twice weekly enhances cardiovascular adaptability (83). In elderly hypertensive patients, resistance training combined with respiratory regulation simultaneously reduces sympathetic activity and enhances blood pressure reduction (86).

High-Intensity Interval Training (HIIT): As a time-efficient alternative, HIIT demonstrates advantages in reducing 24-hour ambulatory blood pressure while effectively improving vascular endothelial function and reducing arterial stiffness (86, 87). For patients aged 65 and older, HIIT exhibits good safety under medical supervision (88).

###### 4.3.1.1.3 Behavioral support

Enhance adherence through health education and self-monitoring (e.g., dietary and weight logging).

Apply cognitive behavioral techniques (e.g., appetite awareness training) to promote healthy behaviors.

Leverage digital health tools (e.g., mobile health apps) to provide continuous feedback and support, reinforcing intervention outcomes (84).

##### 4.3.1.2 Step Two: Pharmacotherapy—adjunct to lifestyle interventions

When lifestyle interventions alone prove insufficient (typically for BMI ≥27 kg/m² with comorbidities like hypertension), pharmacologic weight loss support may be employed. Glucagon-like peptide-1 (GLP-1) receptor agonists represent the most evidence-based class of medications (89, 90).

###### 4.3.1.2.1 GLP-1 receptor agonists

Efficacy: Subcutaneous injection of semaglutide (2.4 mg/week) or tirzepatide (5–15 mg/week) achieves 9–12 kg weight loss and 5–7 mmHg systolic blood pressure reduction over 12 months (90). A 2025 Bayesian network meta-analysis involving 29,506 patients confirmed that GLP-1 receptor agonists reduce weight by an average of 9.0 kg and lower systolic blood pressure by 5.3 mmHg (91). Dual receptor agonists demonstrated superior weight loss effects, reducing weight by an average of 11.0 kg and lowering systolic blood pressure by 6.8 mmHg (91).

Multiple Mechanisms of Benefit: Beyond weight loss, GLP-1 drugs independently improve endothelial function and suppress renin-angiotensin system activity, with effects positively correlated with baseline BMI (92).

Indications and Precautions: Suitable for obese patients with hypertension, type 2 diabetes, or high cardiovascular risk. Liver and kidney function must be assessed, and contraindications evaluated (90). A 2024 meta-analysis revealed that GLP-1 receptor agonists can help patients with post-bariatric surgery weight regain by achieving an additional 7.83 kg loss and improving blood pressure control (93).Oral formulations show similar efficacy but with higher incidence of gastrointestinal adverse events (94).

###### 4.3.1.2.2 Other medications

Orlistat (lipase inhibitor): Aids weight loss but has weak antihypertensive effects (84).

Sympathomimetic weight loss drugs (such as phentermine): Not recommended for hypertensive patients due to potential blood pressure elevation (76).

##### 4.3.1.3 Step Three: Metabolic surgery—advanced option for severe obesity

For severely obese patients with poorly controlled hypertension, metabolic surgery offers significant and sustained blood pressure reduction (95, 96).

###### 4.3.1.3.1 Procedures and efficacy

Roux-en-Y Gastric Bypass (RYGB): Maintains sustained systolic blood pressure reduction of 5–6 mmHg at 5-year follow-up, with hypertension remission rates reaching 35%-51% (96).

Sleeve Gastrectomy: Yields slightly less blood pressure reduction than RYGB but still achieves 4–5 mmHg systolic lowering and medication reduction (96).

Endoscopic Balloon Therapy: As a less invasive option, short-term studies indicate that a 6-month gastric balloon placement reduces body weight by 12% and significantly lowers systolic blood pressure, with particularly pronounced effects in individuals with higher baseline blood pressure (97).

###### 4.3.1.3.2 Long-term benefits and mechanisms

Metabolic surgery sustainably prevents new-onset hypertension. An 8-year prospective study revealed a 71% lower risk of new hypertension in the surgical group compared to controls, with consistent effects across age, gender, and socioeconomic status (95).

The procedure achieves blood pressure reduction through multiple pathways, including weight loss, decreased sympathetic nervous system activity, renin-angiotensin system modulation, and reduced inflammatory markers (98).

###### 4.3.1.3.3 Postoperative management and multidisciplinary collaboration

Long-term monitoring of nutritional status and blood pressure changes is required postoperatively, necessitating coordinated management by a multidisciplinary team encompassing endocrinology, cardiovascular surgery, and nutritional science (99, 100).

#### 4.3.2 Summary and Considerations for Special Populations

Weight management for hypertension should follow a stepwise approach: personalized lifestyle interventions are the first choice; if insufficient, medications such as GLP-1 receptor agonists (GLP-1RAs) may be added to aid weight loss; for severe obesity and resistant hypertension, metabolic surgery may be considered as an effective option. All strategies require multidisciplinary collaboration and long-term follow-up to achieve blood pressure control and reduce cardiovascular risk.

For children and adolescents, weight management should be integrated into routine health examinations, including BMI calculation. Non-hypertensive individuals should aim for healthy weight to prevent hypertension, while hypertensive children should reduce blood pressure through weight loss (79).

Additionally, maintaining a healthy weight significantly reduces the risk of metabolic-associated fatty liver disease, which is closely linked to insulin resistance and cardiovascular risk (101). Obesity has also been identified as a major preventable factor for gout, highlighting its broad metabolic impact (43). Stratifying obesity levels can further optimize cardiovascular prevention strategies and enable more precise interventions (102).

### 4.4 Stress management and mental health

Psychological stress and mental health disorders can contribute to the development and exacerbation of hypertension. PTSD and Hypertension: Post-Traumatic Stress Disorder (PTSD) has been associated with an increased incidence of hypertension, highlighting the physiological impact of chronic psychological distress on the cardiovascular system (41). This connection underscores the importance of integrating mental health support into hypertension prevention and management programs. Depression and Cognitive Decline: Depression is a known risk factor for Alzheimer’s disease (103), and while multidomain interventions may have limited impact on depression, addressing mental health remains crucial for overall well-being and potentially for mitigating cognitive decline (104). Strategies that promote mental well-being, such as mindfulness, relaxation techniques, and adequate sleep, indirectly support blood pressure regulation by reducing sympathetic nervous system activity and inflammatory responses.

Psychological interventions, including but not limited to mindfulness, meditation, proper sleep habits, social support, and digital tools, have been demonstrated to reduce blood pressure and enhance overall health. A growing body of research has demonstrated the efficacy of mindfulness as a psychological intervention, with studies indicating its potential to reduce blood pressure and enhance mental health outcomes. A growing body of research suggests that practices such as mindfulness exercises, meditation, yoga, and deep breathing techniques can effectively alleviate stress, thereby aiding blood pressure control. Maintaining optimal sleep habits is imperative for effective blood pressure management. Sleep disorders, including insomnia and sleep apnea, have been demonstrated to exhibit a notable correlation with hypertension. Maintaining healthy sleep patterns, ensuring adequate sleep duration (7–9 hours), and avoiding prolonged late-night activities are recommended. Digital health interventions, encompassing wearable devices, mobile applications, and online support systems, have emerged as ancillary instruments to assist patients in blood pressure monitoring, behavioral change tracking, and the provision of psychological support (76).

### 4.5 Tobacco and alcohol avoidance

Tobacco cessation and alcohol moderation are cornerstone interventions for cardiovascular health. Smoking and certain metabolic factors significantly increase the risk of early-onset myocardial infarction (105). These habits directly contribute to endothelial damage, arterial stiffening, and increased blood pressure, making their avoidance fundamental to hypertension prevention and control. It is noteworthy that e-cigarettes have increasingly become a subject of interest among smokers as a potential alternative to traditional cigarettes. The effects of e-cigarettes on the cardiovascular system represent a complex and ongoing area of research. Existing evidence suggests that e-cigarette use is associated with short-term changes in heart rate and blood pressure. For instance, a systematic review and meta-analysis indicated that acute e-cigarette exposure is linked to elevated heart rate and blood pressure, though its impact on systolic and diastolic blood pressure is less pronounced compared to traditional cigarettes (106). Another study also indicated that e-cigarette users consume nicotine more frequently, and nicotine intake is significantly associated with increased blood pressure and heart rate (107). Current research does not provide sufficient evidence to support a direct link between e-cigarette use and cardiovascular disease, cardiac dysfunction, or remodeling. However, e-cigarettes may indirectly affect cardiovascular health by influencing endothelial function and hemodynamics (108). Current research has several limitations, including small sample sizes, inconsistent study designs, and a lack of long-term follow-up data. Therefore, future studies should expand sample sizes, control for confounding factors, and conduct long-term follow-ups to better assess the long-term effects of e-cigarettes on the cardiovascular system. In summary, no tobacco product has been proven to be safe and risk-free.

Tobacco and alcohol use recommendations include limiting or avoiding alcohol and strongly advising smoking cessation. Alcohol intake patterns vary by region, encompassing frequency, types, and associated behaviors. Intake levels exhibit substantial regional disparities. In order to achieve optimal cardiovascular health, it is recommended that individuals abstain from alcohol consumption. The recommended daily upper limit for alcohol consumption is two standard drinks for men and one standard drink for women (10 grams of alcohol per standard drink). It is strongly recommended that individuals attempt to cease smoking. It is imperative to implement measures aimed at averting weight gain subsequent to smoking cessation (76).

Lifestyle interventions have been demonstrated to be efficacious in reducing blood pressure, thereby enhancing hypertension control. Of particular significance is the observation that lifestyle modifications have the capacity to engender marked enhancements in cardiovascular health and general well-being. Regardless of genetic predisposition, adopting a healthy lifestyle—including managing body mass index (BMI), improving dietary habits, quitting smoking, moderating alcohol consumption, and reducing sodium intake—can lower systolic blood pressure by 3.5 mmHg and reduce the risk of cardiovascular disease (CVD) by around 30%. Health education interventions can effectively address these factors by reducing salt and fat intake, adjusting dietary habits to prioritize fruit and vegetables, encouraging smoking cessation, limiting alcohol consumption, promoting regular exercise, maintaining a healthy weight and managing stress. These behaviors have a positive effect on blood pressure by regulating visceral fat accumulation, insulin resistance, the renin-angiotensin-aldosterone system, vascular endothelial function, oxidative stress, inflammation and autonomic nervous system activity (84). Lifestyle interventions have been demonstrated to enhance the efficacy of pharmaceutical therapy. In circumstances where drug therapy is necessary, it is recommended that lifestyle adjustments be implemented concurrently. However, the effectiveness of lifestyle support can be hindered by insufficient implementation, impacting overall cardiovascular risk reduction (109).The International Society of Hypertension (ISH) expert committee convened an international team of specialists to develop a policy statement providing practical guidance for healthcare providers and public health programs worldwide. This document has been developed based on the latest evidence and existing guidelines, and has been subject to a joint review and endorsement by the World Hypertension Alliance and the European Society of Hypertension. The text emphasizes that lifestyle interventions are fundamental to the management of hypertension. For individuals with normal blood pressure, these interventions are crucial in preventing the development of hypertension. For individuals diagnosed with hypertension, lifestyle modifications represent the primary therapeutic intervention. In instances where lifestyle modifications prove ineffective in managing blood pressure, the implementation of combined pharmacological therapy is recommended to enhance efficacy. It is imperative to recognize that even in circumstances where the initiation of medication is indicated, the implementation of sustained lifestyle interventions remains of the essence. It is imperative that healthcare professionals receive specialized training in order to engage in lifestyle interventions with patients.

## 5 Novel insights into molecular and physiological mechanisms

Although the benefits of lifestyle interventions for hypertension and its associated complications are well-established, the underlying molecular and physiological mechanisms continue to be an area of active exploration. Recent research has unveiled a range of novel mechanisms that extend beyond traditional pathophysiological models, providing fresh perspectives on how diet and exercise fundamentally regulate blood pressure and organ health.

### 5.1 Novel signaling molecules and regulatory pathways

Moving beyond the classical catecholamine system, emerging research has identified novel endogenous signaling molecules with significant cardiovascular effects. A notable example is 6-nitrodopamine (6-ND), a recently identified endogenous catecholamine that exhibits a potent, nitric oxide (NO)-dependent positive chronotropic effect. The discovery that 6-ND’s activity is abolished in models of NO-deficient hypertension (88) highlights its role within a crucial vasodilatory pathway. This underscores a potential mechanistic link to lifestyle interventions, such as consumption of nitrate-rich vegetables (e.g., beetroot, leafy greens), which are known to enhance NO bioavailability and improve endothelial function (58).

### 5.2 Endothelial dysfunction, oxidative stress, and inflammation: core pathways in diverse forms of hypertension

This triad of pathological processes represents a common final pathway in various forms of hypertension, from drug-induced to salt-sensitive models. Vascular endothelial integrity is the cornerstone of blood pressure homeostasis. Endothelial dysfunction, characterized by diminished nitric oxide (NO) bioavailability and increased oxidative stress, is not only a core mechanism in primary hypertension but also a pivotal driver in other forms of elevated blood pressure. For instance, the hypertension induced by Bruton’s tyrosine kinase inhibitors (BTKis) in hematologic treatments serves as a compelling model. BTKis induce hypertension primarily by inhibiting the PI3K/Akt/eNOS pathway, reducing NO production, and concurrently increasing oxidative stress, leading to eNOS uncoupling and activation of vasoconstrictive pathways like RhoA/ROCK (89). Similarly, a paradigm shift is observed in our understanding of salt-sensitive hypertension (SSHTN), moving from a “renal-centric” to a “vascular-centric” model. In SSHTN, high salt intake directly induces endothelial inflammation and activation, blunting vasodilation and causing a failure to appropriately reduce peripheral vascular resistance post-salt load. This endothelial dysfunction, mediated by reduced NO and increased oxidative stress, is a primary driver of the increased peripheral resistance, independent of volume overload. Furthermore, research highlights significant sex differences, with females being more susceptible to SSHTN, partly due to the protective effects of estrogen on endothelial function and its interplay with the immune system (90).

### 5.3 The crosstalk between metabolism and inflammation

Hypertension is not an isolated hemodynamic disorder but is intricately intertwined with metabolic dysregulation, and this relationship is now established as causal. Large-scale Mendelian randomization studies provide robust genetic evidence for this link, demonstrating that total triglycerides (TG) are the foremost causal risk factor for elevated systolic and diastolic blood pressure. Furthermore, atherogenic lipoprotein particles (such as those in VLDL, IDL, and LDL subfractions) are causally associated with increased pulse pressure. These findings underscore that lipid abnormalities are not merely comorbid conditions but play a direct causal role in elevating blood pressure, highlighting the imperative for integrated management of both blood pressure and metabolism. Lifestyle intervention remains the cornerstone strategy that simultaneously targets both systems, addressing these shared pathological pathways at their root (92).

### 5.4 Vascular remodeling and extracellular matrix regulation

The dynamic equilibrium of the extracellular matrix (ECM) is critical for maintaining vascular elasticity and compliance. The overactivation of the matrix metalloproteinase (MMP) family, particularly MMP-2 and MMP-9, is a direct cause of hypertensive vascular remodeling and stiffening. These enzymes degrade key ECM components like collagen and elastin, disrupt the balance with their tissue inhibitors (TIMPs), and promote vascular inflammation. They interact bidirectionally with the Renin-Angiotensin-Aldosterone System (RAAS), creating a vicious cycle that drives pathological vascular changes and end-organ damage. Notably, many dietary nutrients found in natural foods have been identified as effective MMP inhibitors. Curcumin, quercetin (found in apples and onions), and epigallocatechin gallate (EGCG) from green tea can suppress MMP activity, revealing a molecular mechanism through which a healthy diet and specific nutritional interventions can directly protect vascular structure and function, thereby mitigating hypertension-induced remodeling (91).

### 5.5 Mechanistic links to end-organ damage and therapeutic implications

Sustained hypertension inflicts damage on terminal organs like the heart, brain, and kidneys through the multifaceted mechanisms described above (endothelial dysfunction, oxidative stress, inflammation, RAAS/MMP activation). The choice of antihypertensive therapy can significantly influence this risk based on its mechanism of action. Large-scale real-world evidence indicates that the neuroprotective effect of antihypertensive drugs is closely linked to their ability to penetrate the blood-brain barrier (BBB). Angiotensin II Receptor Blockers (ARBs) that can cross the BBB are associated with a significantly greater reduction in the risk of Alzheimer’s disease and related dementias (ADRD) compared to those that cannot or compared to ACE inhibitors. This underscores the direct impact of central RAAS inhibition and the cerebral microenvironment on cognitive health (93).

Concurrently, the importance of early and aggressive blood pressure management is paramount in high-risk populations, such as children with chronic kidney disease (CKD), to prevent irreversible cardiac remodeling. Clinical trials are actively exploring whether intensive blood pressure control can improve diastolic function and prevent adverse cardiac changes in this vulnerable group, emphasizing that the timing and intensity of intervention are critical for long-term organ protection (94).

In summary, the value of lifestyle interventions extends far beyond the improvement of clinical endpoints. It is deeply rooted in their multi-faceted, network-like beneficial regulation of endothelial function, oxidative stress, inflammation, metabolism, vascular remodeling, and novel signaling pathways. A profound understanding of these effects at the molecular mechanism level will lay a solid foundation for developing more precise and efficient prevention and treatment strategies.

The multifaceted molecular mechanisms through which various lifestyle interventions exert their antihypertensive effects are summarized in **Table 1**.

**Table 1:** Impact of lifestyle interventions on key molecular pathways in hypertension.

Intervention domain	Specific modality	Key molecular & physiological effects	Expected BP reduction (SBP)	Primary references
Dietary Strategies	DASH Diet	Improves endothelial function, reduces oxidative stress, enhances nitric oxide bioavailability	8–14 mmHg	(49, 50)
	Mediterranean Diet	Anti-inflammatory, improves lipid metabolism, enhances endothelial function	4–6 mmHg	(53)
	Sodium Restriction	Reduces vascular resistance, improves renal sodium handling	5–6 mmHg	(47, 54)
	Potassium Supplementation	Counteracts sodium effects, promotes vasodilation	2–4 mmHg	(56)
	Dietary Nitrates (e.g., beetroot)	Increases nitric oxide, improves endothelial function	3–5 mmHg	(58)
Physical Activity	Aerobic Exercise	Improves endothelial function, increases NO bioavailability, reduces sympathetic activity	4–5 mmHg	(71)
	Resistance Training	Enhances vascular compliance, reduces arterial stiffness	6–7 mmHg	(73)
	Isometric Exercise Training (IET)	Improves endothelial function, autonomic regulation, vascular remodeling	5–9 mmHg	(75)
Weight Management	Caloric Restriction + Exercise	Reduces visceral fat, inflammation, RAAS activity, improves insulin sensitivity	~5 mmHg (per 5–10% weight loss)	(76, 82)
	GLP-1 Receptor Agonists	Enhances endothelial function, suppresses RAAS, promotes weight loss	5–7 mmHg	(90, 91)
	Metabolic Surgery	Reduces sympathetic activity, inflammation, improves renal function	4–6 mmHg (sustained)	(95, 96)
Stress Management	Mindfulness & Meditation	Reduces sympathetic nervous system activity, lowers inflammatory markers	2–4 mmHg	(76)
	Sleep Optimization	Improves autonomic balance, reduces nocturnal BP variability	2–3 mmHg	(76)
Tobacco & Alcohol Avoidance	Smoking Cessation	Improves endothelial function, reduces oxidative stress and arterial stiffness	2–4 mmHg	(76)
	Alcohol Moderation	Reduces sympathetic activation, improves baroreflex sensitivity	2–3 mmHg	(76)

The data in the column "Expected BP Reduction (SBP)" represents the reduction range of systolic blood pressure, measured in mmHg.

In the "References" column, we have used the reference numbers as provided in the paper, which correspond to the reference list at the end of your document.

## 6 Integrated and modern approaches to lifestyle intervention

The increasing complexity of chronic disease management, coupled with rapid technological advancements, has propelled the evolution of lifestyle interventions beyond isolated recommendations to integrated, multi-modal, and technologically enhanced approaches. These modern strategies aim to maximize efficacy, improve adherence, and broaden accessibility.

### 6.1 Multidomain interventions

Recognizing the multifactorial nature of many chronic conditions, especially cognitive decline, researchers and clinicians have increasingly advocated for multidomain interventions that concurrently target several modifiable risk factors. Preventing Cognitive Decline: Multi-domain interventions, which typically combine dietary modifications, physical activity, cognitive training, and vascular risk factor management, have demonstrated efficacy in preventing cognitive decline and reducing dementia risk (110–112). These interventions capitalize on the synergistic effects of addressing multiple pathways simultaneously, such as improving cerebrovascular health, reducing inflammation, and enhancing brain plasticity. Such comprehensive approaches are designed to improve overall brain vascular function, thereby supporting cognitive integrity (15). Holistic Brain Health: A digital, multi-modal lifestyle intervention has been specifically shown to promote brain health, indicating the powerful combination of diverse lifestyle elements delivered through modern platforms (113). While some multi-domain interventions have shown limited impact on depression directly (104), their overall benefits for cognitive and physical health are substantial. Brain health clinics are also emerging as specialized centers for managing cognitive impairment, offering integrated care that often incorporates lifestyle counseling (114). Comprehensive Disease Management: For conditions like hypertension and diabetes during pregnancy, comprehensive, multi-faceted interventions are required, often integrating lifestyle components with close medical monitoring (35, 39). Similarly, for individuals after a heart transplant, tailored and comprehensive prevention and rehabilitation guidelines underscore the importance of integrating medical and lifestyle support (25).

### 6.2 Technological advancements and digital health

The digital revolution has profoundly transformed healthcare delivery, offering innovative avenues for implementing and supporting lifestyle interventions. Digital Platforms for Disease Prevention and Management: Digital platforms and mobile health (mHealth) applications are increasingly utilized to facilitate lifestyle modifications. For stroke prevention, digital tools can enhance patient education, monitoring, and adherence to preventive strategies (19). In cardiac rehabilitation programs, digital health interventions have proven effective in promoting weight loss, a key component of cardiovascular recovery (115). For hypertension management, network-based digital interventions have shown significant positive effects, improving blood pressure control through remote monitoring, personalized feedback, and educational content (116). Digital screening also assists in community-based hypertension management, allowing for wider reach and earlier detection (117). These approaches are being validated by real-world applications. For instance, AI-powered personalized recommendations—such as those from the “MyHeart” platform, which correlates real-time dietary records with blood pressure data—have been shown to increase the 6-month blood pressure control rate by 31% (118). Similarly, the integration of remote monitoring, where home blood pressure monitor data is automatically synchronized with community health systems and combined with biweekly nurse follow-up calls, has raised intervention adherence rates to 82% (81). Artificial Intelligence and Machine Learning: The advent of Artificial Intelligence (AI) and Machine Learning (ML) brings unprecedented capabilities to risk prediction and personalized health interventions. AI models are being developed and refined to predict hypertension risk, utilizing vast datasets of clinical, demographic, and lifestyle information (119). Similarly, machine learning techniques are employed to predict hypertension risk in specific populations, such as residents of desert areas in China, allowing for targeted public health interventions (120). These technologies hold immense promise for more precise risk stratification and the delivery of highly individualized lifestyle recommendations. Big Data and Predictive Analytics: The analysis of large-scale datasets, including genetic information, lifestyle patterns, and clinical markers, is leading to new insights. For example, machine learning models can predict hypertension risk based on various factors (120). While lifestyle factors are crucial, some serum biomarkers have shown superior predictive power for lifespan compared to lifestyle scores alone (121), suggesting a future where combined approaches using both lifestyle and advanced biological markers could optimize risk assessment. Furthermore, models like the Singaporean model predict cognitive impairment risk in the elderly (122), highlighting the role of predictive analytics in targeted preventive efforts. Lifestyle factors integrated with cardiovascular metabolic multimorbidity risk prediction provide a more comprehensive assessment (123).

### 6.3 Public health and policy initiatives

Broad-scale public health policies and initiatives are crucial for fostering environments conducive to healthy lifestyles and supporting widespread adoption of beneficial behaviors. Workplace Interventions: The workplace represents a significant setting for health promotion. Comprehensive workplace interventions have demonstrated substantial improvements in blood pressure control among employees (124). Similarly, specific workplace interventions have been shown to reduce the incidence of hypertension, contributing to a healthier workforce (125). Food Industry Reform: Mandatory sodium substitution technologies at the front end (such as replacing 50% of sodium salts with potassium salts) implemented in Canada and Australia resulted in a median reduction of 1.7 mmHg in systolic blood pressure among the population (76). National Programs: Initiatives like the American Target: BP program have played a vital role in improving blood pressure control rates across the United States, demonstrating the effectiveness of coordinated national efforts (126). Evolving Public Health Landscape: The COVID-19 pandemic significantly altered the landscape of public health policy for healthy living, prompting adaptations in how health information and interventions are disseminated and consumed (127). Early Childhood Intervention: School-based curriculum-integrated healthy eating education combined with family involvement reduces adolescent hypertension risk by 34% (10-year follow-up data) (82). This shift has underscored the importance of resilience and adaptability in public health strategies. Lifestyle Medicine Frameworks: The integration of lifestyle medicine principles, which systematically apply evidence-based lifestyle therapeutic interventions as a primary modality to prevent, treat, and often reverse chronic diseases, is gaining traction. This holistic framework is particularly relevant for older adults, aiming to integrate lifestyle factors into comprehensive care plans (128). Addressing Disparities and Underdiagnosis: Despite advancements, challenges persist in diagnosing and managing hypertension in certain populations, such as rural communities (1). Similarly, issues like low education levels being a primary risk factor for mortality (129) highlight the importance of addressing social determinants of health to achieve equitable health outcomes.

### 6.4 Synergy with pharmacotherapy

While lifestyle interventions are powerful on their own, their integration with pharmacological treatments often yields superior outcomes, particularly in managing established hypertension and complex comorbidities. Combined Benefits for Mortality: A combination of healthy lifestyle adherence and the use of antihypertensive medication has been shown to reduce mortality rates more effectively than either approach alone (48). This synergistic effect underscores that lifestyle is not merely an alternative to medication but a complementary and essential component of comprehensive care. Intensified Blood Pressure Lowering: Intensive blood pressure lowering, often achieved through a combination of lifestyle and pharmacological interventions, has been shown to reduce the risk of cognitive impairment, highlighting the neuroprotective benefits of aggressive hypertension management (9). Antihypertensive Treatment and Cancer Risk: Reassuringly, anti-hypertensive treatment has not been found to have a significant association with cancer risk, allowing clinicians to confidently prescribe these medications without undue concern about oncogenic effects (30). Novel Drug Classes: Newer pharmacological agents, such as SGLT-2 inhibitors, initially developed for diabetes, have demonstrated significant cardiovascular and renal protective effects, including in diabetic cardiomyopathy, showcasing how advancements in drug therapy can complement lifestyle efforts in managing complex cardiometabolic conditions (130) While combined hypertension and diabetes intervention may not always significantly reduce CVD events, emphasizing comprehensive risk reduction remains paramount (131). Targeting Residual Risk: Even with medication, residual risks remain. For example, in patients with atrial fibrillation, approximately 30% of residual stroke risk is modifiable through lifestyle interventions (21). This underscores that even when medications are prescribed, ongoing adherence to lifestyle recommendations remains critical for optimal outcomes. Physicians’ advice for secondary prevention becomes more frequent, highlighting the perceived importance of ongoing intervention (132). However, in China, there is an underutilization of secondary prevention medications for cardiovascular diseases, indicating gaps in care delivery (133).

### 6.5 Key considerations and challenges

Although lifestyle interventions offer a novel approach to the prevention, treatment and reversal of hypertension, multidisciplinary collaboration is required between cardiologists, nutritionists, exercise specialists and psychologists. Clinicians must master systematic lifestyle intervention techniques and follow actionable pathways to provide long-term, sustained guidance and support patients in making behavioral changes. Adherence and Support: Behavioral change is influenced by patients’ values, their social environment, and psychological factors. Without the necessary internal motivation, it is difficult for patients to maintain a lifestyle change in the long term, even if they initially commit to it. Insufficient lifestyle support is a major factor impacting cardiovascular risk reduction (109). Effective strategies require continuous support, education, and patient engagement. As key agents in lifestyle change, physicians face a ‘double whammy’ when their own poor habits undermine both their credibility and their ability to provide support. Most clinicians lack the necessary skills for lifestyle interventions, particularly motivational interviewing (encouraging patients to explore their motivations, resolve conflicts and increase their self-efficacy) and cognitive behavioral therapy (changing unhealthy thought patterns to encourage long-term behavior change). This gap can significantly reduce the effectiveness of lifestyle interventions. Individualization: A one-size-fits-all approach is often insufficient. When developing personalized plans, planners and implementers must communicate thoroughly to clarify desired outcomes. Planners should motivate patients to commit to change while fully considering individual patient characteristics and preferences. This not only enhances patient adherence but also maximizes the effectiveness of lifestyle interventions. Personalized exercise prescriptions, for instance, are more effective in lowering blood pressure (66). Similarly, managing conditions like pregnancy-induced hypertension requires long-term, individualized strategies (39), and post-cardiac transplant patients need customized prevention and rehabilitation plans (25). Social Determinants of Health: The limited availability of regular outpatient clinics makes it difficult to conduct in-depth lifestyle assessments and counselling. The current medical insurance system offers limited reimbursement for lifestyle interventions, which makes it challenging to sustain such programs. Socioeconomic factors, such as low education, significantly impact health outcomes (129). Access to healthy foods, safe environments for physical activity, and healthcare resources varies, creating health disparities. Community-based and digital screening initiatives for hypertension can help bridge some of these gaps, particularly in underserved areas. Implementation will encounter multiple interrelated challenges, including insufficient digital literacy, technological limitations, resource scarcity, and low stakeholder engagement. Addressing these challenges requires a multi-pronged strategy encompassing enhanced digital literacy training, improved technical support systems, robust data management frameworks, and deepened collaboration between healthcare systems and digital health initiatives—undoubtedly demanding increased human and material resources (117). Emerging Environmental and Occupational Risk Factors: Environmental factors are increasingly recognized as contributors to chronic diseases. Air pollution, for example, is positively associated with colorectal cancer risk (33). Exposure to lead also correlates with an increased risk of hypertension (134). Per- and polyfluoroalkyl substances (PFAS), while showing a weak association with blood pressure (134), can impact kidney function (135), underscoring the need to consider environmental toxicants in a comprehensive health assessment. Occupation itself can be a risk factor for type 2 diabetes (136).

## 7 Clinical implications and future directions

### 7.1 The evidence synthesized in this review has direct implications for clinical practice

To facilitate the translation of this evidence into actionable clinical strategies, a framework for personalized lifestyle prescription is provided in **Table 2**.

**Table 2:** Evidence-based personalized prescription for lifestyle medicine in hypertension.

Intervention Domain	Core recommendation	Personalization & special considerations	Key evidence / expected benefit
Dietary Strategies	Adopt evidence-based patterns: DASH or Mediterranean diet. Restrict sodium to <2.3g/day. Increase potassium-rich foods.	• Diabetes: DASH may have a limited BP-lowering effect; focus on overall cardiometabolic health. • CKD: Prioritize plant-based diets; monitor potassium under medical guidance. • Salt-Sensitive HTN: Emphasize strict sodium restriction. • Cultural Preferences: Adapt recommended dietary patterns to align with cultural food traditions.	• DASH: Reduces SBP by 8–14 mmHg (49, 50). • Mediterranean Diet: Reduces CVD risk and SBP by 4–6 mmHg (53). • Sodium Restriction: Reduces SBP by 5–6 mmHg [47, 76].
Physical Activity	≥150 mins/week moderate aerobic exercise OR 75 mins/week vigorous aerobic exercise. Add resistance training 2 days/week.	• Elderly/Frailty: Focus on balance and resistance training to prevent falls; consider respiratory-regulated resistance training. • Arthritis: Prefer low-impact activities (swimming, cycling). • Time-Constrained: Recommend High-Intensity Interval Training (HIIT). • Resistant HTN: Isometric Exercise Training (IET) can be particularly effective.	• Aerobic Exercise: Reduces SBP by 4–5 mmHg (71). • Resistance Training: Reduces SBP by ~7 mmHg in the elderly (73). • IET: Can reduce SBP by 5–9 mmHg (75).
Weight Management	Target 5-10% weight loss through combined dietary and exercise interventions.	• BMI ≥27 with Comorbidities: Consider GLP-1 receptor agonists (e.g., semaglutide) as an adjunct. • Severe Obesity/Resistant HTN: Evaluate for metabolic surgery (e.g., Roux-en-Y Gastric Bypass). • Children/Adolescents: Integrate family-based behavioral interventions.	• Lifestyle Intervention: ~1 mmHg SBP reduction per kg lost (82). • GLP-1 RAs: ~5-7 mmHg SBP reduction with 9-12 kg weight loss (90, 91). • Metabolic Surgery: Sustained SBP reduction of 4-6 mmHg and high remission rates (95, 96).
Stress Management & Mental Health	Practice mindfulness, meditation, or yoga. Ensure 7-9 hours of quality sleep per night.	• PTSD/Depression: Integrate mental health screening and support; these conditions are linked to increased hypertension risk (41, 103). • Shift Workers: Focus on sleep hygiene and circadian rhythm alignment. • Digital Tools: Use apps for guided meditation and sleep tracking to improve adherence.	• Improved autonomic balance and reduced sympathetic activity. • Expected SBP reduction of 2-4 mmHg (76).
Tobacco & Alcohol Avoidance	Complete smoking cessation. Limit alcohol to ≤2 drinks/day (men) or ≤1 drink/day (women).	• E-cigarette Users: Counsel that no tobacco product is risk-free; e-cigarettes can acutely increase heart rate and BP (106, 107). • High-Risk Drinkers: Implement structured cessation programs. • Post-Cessation: Provide dietary support to prevent weight gain.	• Smoking cessation improves endothelial function and reduces arterial stiffness. • Alcohol moderation prevents alcohol-induced BP elevation. • Combined healthy lifestyle changes can reduce SBP by ~3.5 mmHg and CVD risk by ~30% (76, 84).

Structured Patient Assessment: Clinicians should routinely and systematically assess lifestyle factors during every encounter with hypertensive patients or those at risk. Simple tools like the American Heart Association’s Life’s Essential 8 or taking a “lifestyle history” can be integrated into electronic health records to facilitate this. Personalized Prescription for Lifestyle Medicine: Just as with pharmacology, lifestyle interventions should be personalized. This involves: Exercise Prescription: Specifying type (aerobic, resistance, HIIT), frequency, intensity, and time, tailored to the patient’s fitness, comorbidities, and preferences. Dietary Counseling: Moving beyond generic “eat healthy” advice to recommending specific evidence-based patterns like DASH or Mediterranean diet, with practical guidance and, if possible, referral to a dietitian. Shared Decision-Making: Discussing the robust evidence for lifestyle interventions with patients, including their synergistic effects with medication and potential to reduce pill burden, can enhance motivation and adherence. Utilization of Digital Health Tools: Clinicians should advocate for and utilize digital health platforms (e.g., apps for BP monitoring, diet tracking, tele-rehabilitation) to extend support beyond the clinical setting and improve long-term engagement. Multidisciplinary Care Model: Effective hypertension management requires a team-based approach. Cardiologists and general practitioners should work closely with nurses, dietitians, physiotherapists, and psychologists to deliver comprehensive lifestyle counseling and support. Policy Advocacy: Healthcare providers should advocate for broader public health policies that support lifestyle medicine, such as sodium reduction in processed foods, creation of safe spaces for physical activity, and insurance reimbursement for structured lifestyle intervention programs. While implementing the current evidence into practice is paramount, the field of hypertension management continues to evolve. The next frontier lies in moving beyond standardized approaches to embrace precision prevention and to solve the challenge of long-term maintenance of healthy lifestyles.

### 7.2 Future directions: precision prevention and long-term maintenance

The future of hypertension management lies in moving beyond one-size-fits-all approaches to precision prevention and developing effective strategies for long-term maintenance of healthy behaviors.

#### 7.2.1 Early identification of high-risk populations

Polygenic Risk Score (PRS): Initiating intensive lifestyle interventions a decade in advance for individuals identified as having high genetic risk (top 10% PRS) can reduce the conversion rate to hypertension by 47% (76). Application of Metabolic Biomarkers: For individuals with serum uric acid >380 μmol/L or abnormal cystatin C levels, weight loss interventions are recommended even in the presence of normal blood pressure, facilitating preemptive management (82).

#### 7.2.2 Evidence updates for maintenance strategies

Periodic Booster Interventions: Centralized lifestyle courses conducted every 4 months (including group exercise and dietitian counseling) can address behavioral fatigue and reduce the 5-year weight rebound rate to 22% (118). Preventive Use of GLP-1 Receptor Agonists: For prediabetes populations with a BMI ≥27 kg/m², low-dose semaglutide (0.5 mg/week) combined with lifestyle intervention can reduce the risk of developing hypertension by 62% (76).

## 8 Conclusion

Hypertension stands as a formidable global health challenge, intricately linked to a myriad of cardiometabolic and cognitive disorders. The complex interplay between hypertension and these conditions underscores the importance of comprehensive, personalized interventions for prevention and management. Lifestyle interventions—including dietary modifications, physical exercise, weight management, and stress reduction—form the foundation for effectively preventing and controlling hypertension and its complications. Substantial evidence demonstrates that such interventions significantly lower blood pressure, improve cardiovascular health, and reduce the risk of complications such as cardiovascular disease, chronic kidney disease, and cognitive decline. Notably, the effectiveness of these interventions increases substantially when tailored to individual patient characteristics, preferences, and needs. Personalized approaches—such as customized exercise prescriptions and dietary recommendations—further ensure long-term adherence and enhance treatment outcomes. Beyond individual interventions, comprehensive risk assessment tools such as the American Heart Association’s Life’s Essential 8 and the Brain Care Score have been developed to provide a holistic framework for cardiovascular and cognitive health promotion (137, 138). These tools underscore the importance of integrating multiple lifestyle and health metrics in clinical practice to optimize patient outcomes. Clinicians should routinely assess patients’ lifestyle habits using simple tools and provide structured, patient-centered counseling, leveraging digital health platforms for long-term support. Policymakers should prioritize national hypertension control programs, implement policies for reducing sodium in processed foods, and create environments that promote physical activity. Future research should focus on long-term effectiveness of digital interventions, cost-effectiveness analyses, and implementation strategies in low-resource settings. In summary, managing hypertension through integrated approaches combining lifestyle interventions, clinical care, and public health policies represents a critical pathway to reducing the global burden of hypertension and its complications.

In conclusion, a robust and sustained commitment to lifestyle modifications represents the most potent and accessible strategy for individuals and public health systems alike in the ongoing battle against hypertension and its pervasive consequences. Embracing lifestyle as a primary therapeutic modality is not just about managing a single condition; it is about cultivating a paradigm of health that promotes longevity, enhances quality of life, and mitigates the intertwined burdens of cardiometabolic and cognitive decline.
